# Wideband Circularly Polarized Millimeter Wave Hemispherical Dielectric Resonator Antenna

**DOI:** 10.3390/mi14020436

**Published:** 2023-02-12

**Authors:** Meshari D. Alanazi, Salam K. Khamas

**Affiliations:** 1 Electrical Engineering Department, Jouf University, Sakaka 72388, Saudi Arabia; 2 Communications Research Group, Department of Electronic and Electrical Engineering, The University of Sheffield, Mappin Street, Sheffield S1 3JD, UK

**Keywords:** circularly polarized, DRA, mm-wave band, hemispherical DRA

## Abstract

A novel approach is proposed to design a circularly polarized (CP) hemispherical dielectric resonator antenna (DRA) with a wide axial ratio (AR) bandwidth by incorporating an additional dielectric substrate between the antenna and the ground plane. This is in addition to the lower feeding substrate that is located between the ground plane on one side and the feeding microstrip line on the other side. Adding another substrate on top of the ground plane provided an additional degree of freedom in the design that facilitated the achievement of ab 18% AR bandwidth. In addition, an integrated hemispherical DRA and perforated substrate configuration was utilized to achieve optimum effective substrate permittivity and overcome the DRA alignment and assembly challenges while maintaining the achieved wide CP bandwidth. A close agreement was achieved between measurements and simulations.

## 1. Introduction

Dielectric resonator antennas are capable of efficiently replacing conventional metallic antennas, especially at the millimeter wave (mmWave) frequencies, due to the absence of ohmic and surface wave losses as well as other appealing advantages [1]. One of the most common DRA feeding mechanisms at the mmWave band is the utilization of a slot aperture to couple the energy to the resonator [2]. This feeding method can be applied conjointly with a coplanar waveguide, microstrip line, or substrate integrated waveguide (SIW) [3]. However, CP radiation resists multipath interference and reduces any polarization mismatch. Furthermore, a CP wave is less sensitive to weather changes and mobility than a linearly polarized (LP) wave. Further, the CP radiation is less sensitive to the alignment requirements between the transmitting and receiving antennas [4]. As a result, several techniques have been reported to efficiently design circularly polarized DRAs, such as various feeding slots shapes [5,6,7,8,9], that also have been utilized in the design of single and multi-point-fed DRAs [10,11,12]. In addition, novel DRA shapes have been proposed to achieve an improved AR bandwidth such as trapezoidal [13], stair-shaped rectangular [14], multi-element rectangular [15], and cross-shaped DRAs [16].

Moreover, several studies have focused on the design of mmWave CP DRAs using a cross-slot feeding [17,18,19,20]. For example, a rectangular DRA has been reported with respective impedance and axial ratio bandwidths of 17% and 12.8%, in conjunction with a gain of 5 dBic at 20 GHz as well as respective impedance and AR bandwidths of 15.2%, 5% and a gain of 8 dBic at 26 GHz [17]. Another study has proposed a layered rectangular DRA configuration with impedance and AR bandwidths of 36.5% and 13.75%, respectively, in combination with a high gain of 12 dBic [18]. In addition, flower-shaped DRA has been reported with respective impedance and AR bandwidths of 33.8% and 5%, with a gain of 9.5 dBic at 30 GHz [19]. An alternative approach has been proposed in [20], in which a frequency-selective surface (FSS) superstrate was utilized with a rectangular DRA to achieve impedance and CP bandwidths of 8.26% and 3%, respectively, with a gain of 8.5 dBic at 30 GHz. Other studies have proposed DRA designs to generate CP radiation at 60 GHz [21,22]. A cylindrical DRA fed using a cross-slot with half-mode surface integrated waveguide provided impedance and axial ratio bandwidths, as well as gains of 4.5%, 4%, and 5.5 dBic, respectively [21]. Furthermore, a rectangular slot fed substrate-integrated cylindrical DRA with a loading patch on top to generate the CP radiation has been reported to have impedance and AR bandwidths of 9.33%, 1.35%, respectively, and a gain of 7.15 dBic [22].

However, circularly polarized hemispherical DRAs have only been considered earlier at lower frequencies using various feeding techniques [23,24,25,26,27]. Hemispherical DRAs are usually reported with narrow CP bandwidths, since the DRA offers zero degrees of freedom, which limits the options when designing an antenna with a reasonably wide AR bandwidth. For example, grounded parasitic patches have been utilized in a combination of conformal strips or rectangular feeding slots with CP bandwidths of 2.4% and 3.4%, respectively [23,24]. Further studies have considered spiral, shorted annular rings, and U-shaped slots in the design of hemispherical CP DRAs with an AR bandwidth range of 2.6–4.5% [25,26]. To the best of the authors’ knowledge, a hemispherical DRA with the widest AR bandwidth of 6% was achieved by utilizing an annular ring feeding slot that was directly soldered to a coaxial cable with a backing hemispherical metal cavity at 2 GHz [27]. However, the utilization of the coaxial cable feed with a backing cavity is impractical at the mmWave frequency range due to the physically small antenna dimensions. It should be noted that a mmWave CP hemispherical DRA has not been reported in the literature. The advantage of a hemispherical over rectangular, cylindrical and other shapes is that the boundary between the dielectric and air is more straightforward, allowing for the derivation of closed-form equations.

In this article, a mmWave hemispherical DRA is proposed with a CP bandwidth that is significantly wider than those attained at low frequencies. The enhanced CP bandwidth was achieved by incorporating an additional dielectric substrate. Two configurations were considered. In the first, a solid top substrate was considered, and in the second an integrated DRA-perforated substrate was utilized with a CP and impedance bandwidths of ∼18%. This achieved wide AR and impedance bandwidths, combined with a high broadside gain of ∼7 dBic at 26 GHz. The prototypes were fabricated and measured, with close agreement between simulated and experimental results. The simulations wee conducted using CST microwave studio.

## 2. Antenna Design and Analysis

### 2.1. Circularly Polarized Antenna

When a plane radio wave propagates over a long distance, it has no field components along the travelling z-direction, and thus is in the form of a transverse electromagnetic (TEM) plane wave. The instantaneous field of such a plane wave can be expressed as:

(1) $$E=E_{x}+E_{y}$$

where the $E_{x}$ and $E_{y}$ components are specified as:

(2) $$E_{x}=E_{xm}\cos\left(\omega t+k_{\mathrm{oz}}+\phi_{x}\right)$$

(3) $$E_{y}=E_{ym}\cos\left(\omega t+k_{\mathrm{oz}}+\phi_{y}\right)$$

where $E_{xm}$ and $E_{ym}$ are the maximum values and $k_{0z}$ is the free-space wavenumber defined as $k_{0z}$= 2π/λ. The radiated wave’s polarisation can be classified into linear, circular, or elliptical based on the direction of its electric field vector *E*. When z = 0, Equation (3) can be simplified as [28]:

(4) $$E_{x}=E_{xm}\cos\left(\omega t+\phi_{x}\right)$$

(5) $$E_{y}=E_{ym}\cos\left(\omega t+\phi_{y}\right)$$

The polarization is circular only when both $E_{x}$ and $E_{y}$ field components have the same magnitude and in quadrature, i.e.,

(6) $$E_{xm}=E_{ym}=E_{m}$$

(7) $$\Delta \phi =\phi_{y}-\phi_{x}=\pm \frac{\pi }{2}+2n\pi ,\;n=0,1,2,\ldots$$

When $\phi_{y}-\phi_{x}=\pi /2$, Equations (4) and (5) can be expressed as:

(8) $$E_{x}=E_{m}\cos\left(\omega t+\phi_{x}\right)$$

(9) $$E_{y}=E_{m}\cos\left(\omega t+\phi_{x}+\frac{\pi }{2}\right)=-E_{m}\sin\left(\omega t+\phi_{x}\right)$$

**Figure 1:**
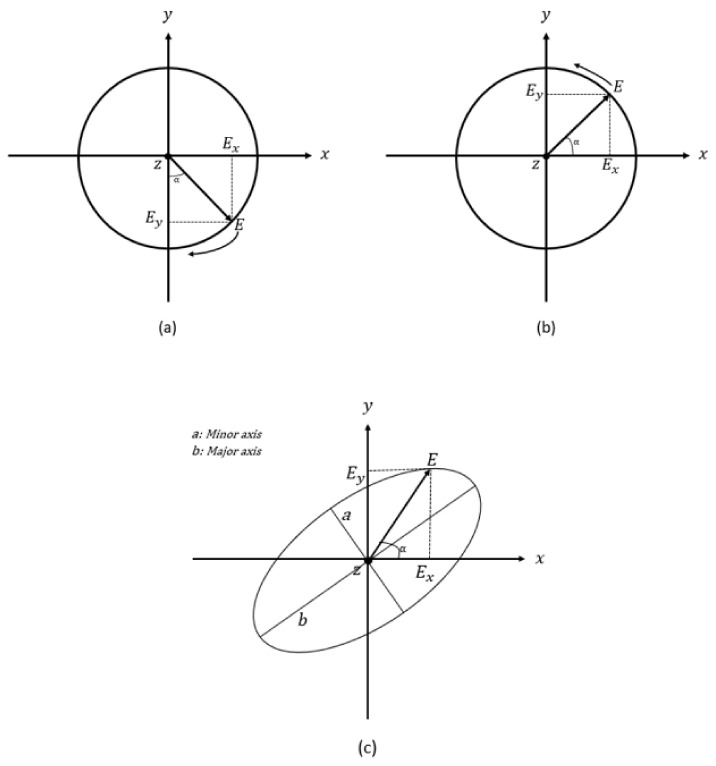
E-field vector of (**a**) Right-hand circular polarisation and (**b**) Left-hand circular polarisation (**c**) Elliptical polarisation.

It is well-known that the magnitude of the electric field vector is constant, and the direction varies with time. Accordingly, the electric field vector rotates in a circle in the xy plane, i.e., it rotates along the propagation direction, as illustrated in Figure 1. Furthermore, in the case of circular polarisation, when the rotation of the electric field vector is clockwise, the polarization sense is defined as a Left-Hand Circular Polarisation (LHCP), as illustrated in Figure 1a. When the electric field vector rotates counterclockwise, as illustrated in Figure 1b, the polarization sense is defined as Right-Hand Circular Polarisation (RHCP).

The quality of the circular polarisation was assessed using the axial ratio (AR) parameter, which is calculated by dividing the maximum by the minimum electric field strengths that correspond to the maximum and minimum radii of the ellipse, respectively, as illustrated in Figure 1c. Therefore, the mathematical expression of the axial ratio is:

(10) $$\mathrm{AR}=20\log_{10}\left(\frac{E_{\max}}{E_{\min}}\right)$$

where |E|(max/min)$=\sqrt{\frac{1}{2}\left(E_{x}^{2}+E_{y}^{2}\pm \sqrt{E_{x}^{4}+E_{y}^{4}+2E_{x}^{2}E_{y}^{2}\cos2\Delta \phi }\right)}$. From the above equation, it can be noted that when $E_{x}$ and $E_{y}$ have the same magnitudes and a phase difference of $90^{\circ }$, then the $E_{\max}=E_{\min}$ and AR=0dB, which means the wave’s polarisation is circular. Practically, AR≤3dB is the acceptable level of AR in which a circular polarisation is considered to be achieved, i.e., a linear polarisation exists when AR>3dB.

### 2.2. Antenna Configuration

**Figure 2:**
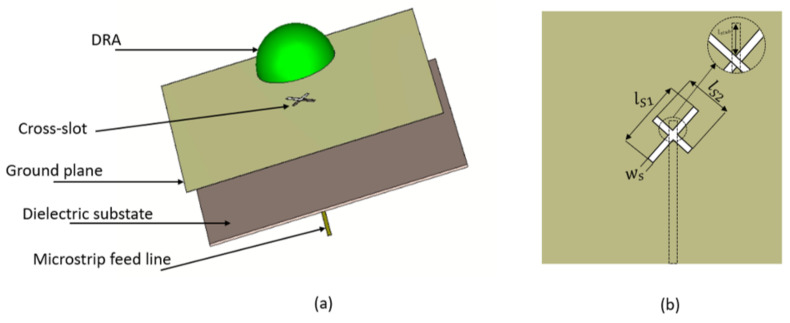
Antenna configuration (**a**) 3D view (**b**) Feed network.

As illustrated in Figure 2, the hemispherical DRA is placed above on the top of a ground plane with an etched cross-slot that has respective arm lengths of $l_{s_{1}}$ and $l_{s_{2}}$. The cross-slot has equal arm widths of Ws. An Alumina DRA has been assumed with a radius of *r* = 3.8 mm and dielectric constant of $\varepsilon_{r}$ = 9.9. The unequal cross-slot arm lengths excite the required orthogonal degenerate modes that generate the CP wave. A Rogers substrate, Ro3006, has been utilized with a dielectric constant of $\varepsilon_{r_{1}}$ = 6.5, loss tangent of tanδ = 0.002 and a thickness of $h_{1}$ = 0.3 mm. At the lower side of the substrate, a microstrip line is placed that extends beyond the cross-slot’s centre to form a matching stub with a length of $l_{stub}$.

**Figure 3:**
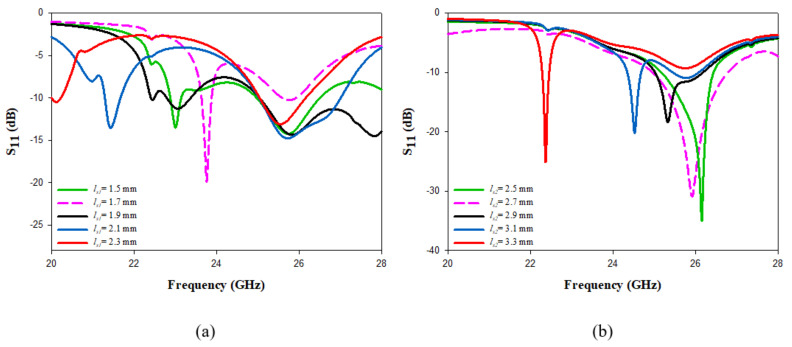
The effects of changing the cross-slot arm’s length on the reflection coefficient (**a**) $l_{s1}$ (**b**) $l_{s2}$.

**Figure 4:**
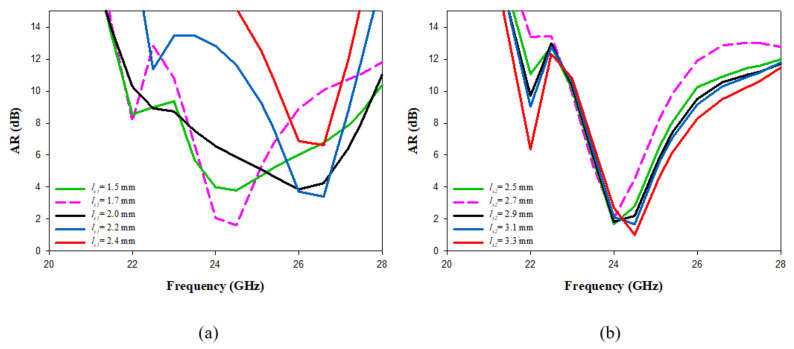
The effect of varying the cross-slot arm length on the axial ratio (**a**) $l_{s1}$ (**b**) $l_{s2}$.

A parametric sweep study was carried out to investigate the effect of different cross-slot arm’s lengths on the input impedance, gain, and CP bandwidth. Only one parameter changed at a time, while the rest were fixed. The variation in the impedance bandwidth as a function of the lengths of the cross-slot arms is illustrated in Figure 3, where it is evident that changing the arm lengths can significantly affect the excited resonance mode, which impacts the impedance bandwidth. The cross-slot arm length, $ls_{1}$ changed from 1.5 mm to 2.4 mm, while the other slot dimensions were kept constant at $l_{s2}$ = 3 mm and $w_{s}$ = 0.35 mm. When $l_{s1}$ = 1.5, 1.7, 1.9, 2.1 and 2.3 mm, the DRA achieved impedance bandwidths of 5, 1.2, 11.3, 9.2 and 4.6%, respectively. However, the CP was only achieved when $l_{s1}$ = 1.7 mm, as demonstrated in Figure 4a. Next, the cross-slot arm’s length, $l_{s2}$, changed over a range of 2.5 mm to 3.3 mm by a step size of 0.2 mm, while $l_{s1}$ = 1.7 mm. Again, the arm length has a considerable impact on the resonance frequency, as well as the impedance and CP bandwidths. The CP operation was attained at all the aforementioned chosen values of $l_{s2}$, with the widest CP bandwidth attained when $l_{s2}$ = 3.1 mm. Therefore, the optimum return loss and CP bandwidths of 8.3% and 3.6% wee achieved when $l_{s1}$ = 1.7 mm and $l_{s2}$ = 3.1 mm and with a stub length of 0.5 mm. It should be noted that these cross-slot dimensions excited the following DRA resonance modes; $TE_{311}$ and $TE_{112}$ at 23 GHz and 26 GHz, respectively [29].

## 3. Incorporating an Additional Dielectric Substrate Layer

**Figure 5:**
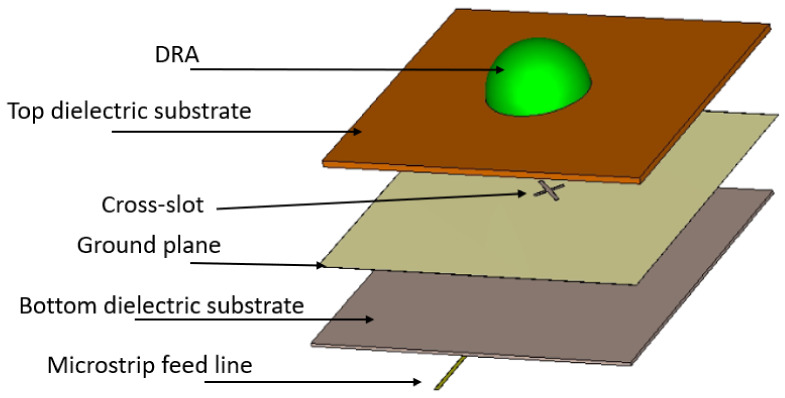
3D view of the proposed configuration using additional substrate with the same feed network.

The configuration of Figure 2 was modified by incorporating an additional dielectric substrate between the DRA and ground plane, as illustrated in Figure 5. The added substrate offers another degree of freedom, which allows for more flexibility and control over the design to achieve a wider CP bandwidth. The substrate has a dielectric constant of $\varepsilon_{r_{2}}$ and a thickness of $h_{2}$, which can be optimized to provide the required CP and impedance bandwidths. The added substrate has a size of 20 mm × 25 mm, which is identical to that of the ground plane and the lower feeding substrate to avoid any potential misalignment in the configuration.

### 3.1. Parametric Study of the Inserted Dielectric Substrate Layer

**Figure 6:**
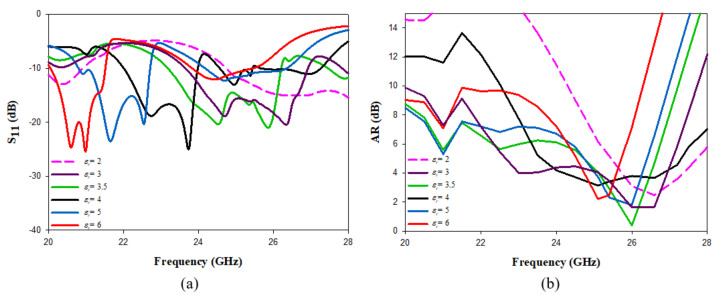
The effect of changing $\varepsilon_{r_{2}}$ on (**a**) reflection coefficient (**b**) axial ratio.

The impact of changing $\varepsilon_{r_{2}}$ was investigated, as demonstrated in Figure 6 when the height of the added top substrate was maintained at $h_{2}$ = 0.6 mm and the examined dielectric constant range was 2 ≤ $\varepsilon_{r_{2}}$≤ 6. As expected, the resonance frequency and impedance matching bandwidth reduced when $\varepsilon_{r_{2}}$ is increased. However, overlapping between the impedance and AR bandwidths was only achieved when $\varepsilon_{r_{2}}$ = 2, $\varepsilon_{r_{2}}$ = 3 and $\varepsilon_{r_{2}}$ = 3.5. The overlap covers frequency ranges of ∼25.8–27.4 GHz, 24.5–27.3 GHz and 24.7–26.2 GHz in the cases of $\varepsilon_{r_{2}}$ = 2, $\varepsilon_{r_{2}}$ = 3 and $\varepsilon_{r_{2}}$ = 3.5, respectively.

**Figure 7:**
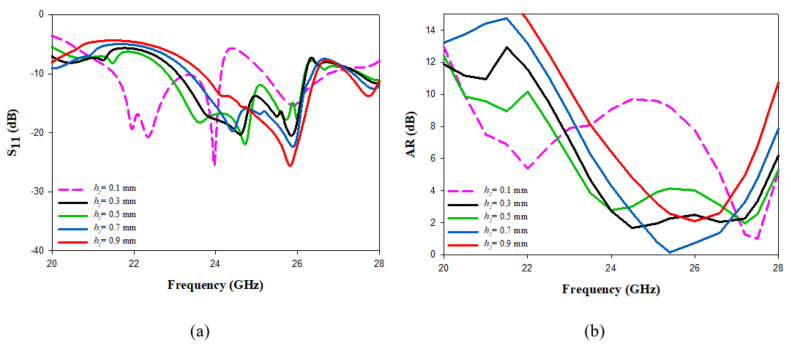
The effect of changing $h_{2}$ on (**a**) reflection coefficient (**b**) axial ratio.

Next, $h_{2}$ was varied from 0.1 to 0.9 mm, as illustrated in Figure 7 when $\varepsilon_{r_{2}}$ = 3, where it can be observed that increasing $h_{2}$ results in a reduction in the impedance bandwidth. For example, an $S_{11}$ bandwidth of 18% was achieved when $h_{2}$ = 0.1 mm compared to 11% when $h_{2}$ = 0.9 mm. Moreover, the widest CP bandwidth of 13.7% was achieved when $h_{2}$ = 0.3 mm, as demonstrated in Figure 7b. Therefore, the ideal top substrate parameters were $\varepsilon_{r_{2}}$ = 3 and $h_{2}$ = 0.3 mm, as they provide impedance and AR bandwidth of 12.5% and 13.7%, respectively, with an overlapping bandwidth of 9%. These bandwidths are considerably higher than those achieved without adding the substrate, where the optimum impedance and AR bandwidths were 8.3% and 3.6%, respectively. The wider bandwidths that were can be attributed to the use of an additional substrate with a lower dielectric constant between the DRA and ground plane. Hence, the electric fields are obliged to pass through the low-permittivity regions, which increases the bandwidth.

**Figure 8:**
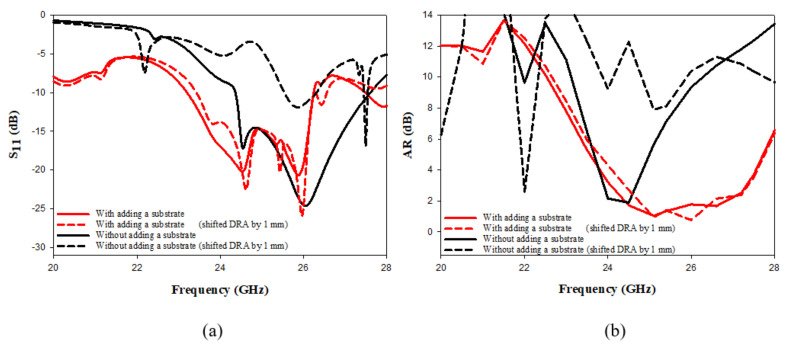
The impact of DRA misalignment on the performance with/without adding the additional substrate (**a**) reflection coefficient (**b**) axial ratio.

As well as improving the CP bandwidth, the inserted dielectric substrate considerably reduces the impact of any misalignment between the DRA and the cross-slot, as demonstrated in Figure 8. For example, a significant change in the performance is evident when the DRA position is shifted by 1 mm with respect to the feeding slot at the absence of the top substrate. However, this impact is almost eliminated when a substrate is added between the DRA and the feeding slot. As mentioned earlier, at mmWave frequencies, a slight misalignment in the DRA position can have a significant impact on the measurement accuracy. Hence, the incorporation of additional substrate represents a simple approach to address these problems.

### 3.2. Measurements of a Prototype with the Additional Solid Dielectric Substrate

In practice, the theoretically identified optimal combination of $\varepsilon_{r_{2}}$ and $h_{2}$ was commercially unavailable. As a result, a Rogers dielectric substrate, Ro4350C, that offers the closest combination of $\varepsilon_{r_{2}}$ = 3.45 and $h_{2}$ = 0.35 mm, with tan δ = 0.0037, was utilized. However, the DRA and feeding substrate dimensions were kept the same as those mentioned in Section 3.1, i.e., a DRA radius and dielectric constant of *r* = 3.8 mm and $\varepsilon_{r}$ = 9.9, respectively. Similarly, the optimized lengths of the cross-slot arms of 1.7 mm and 3.1 mm were maintained, with the same stub length of $l_{stub}$ = 0.5 mm. The introduction of an additional substrate layer resulted in a marginal increase in the configuration’s profile, which can be justified considering the achieved improvement in the CP and impedance bandwidths.

**Figure 9:**
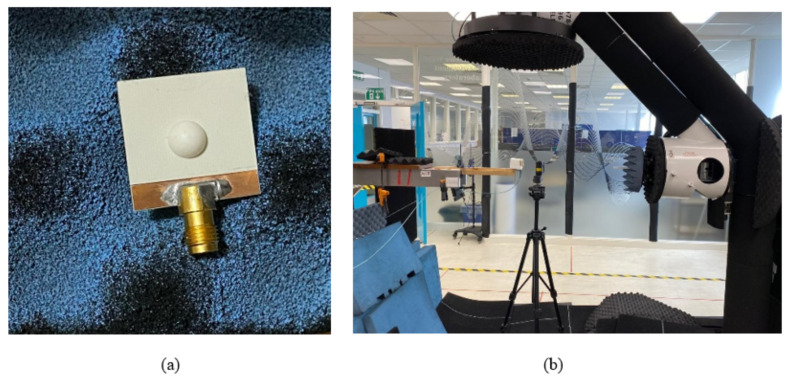
Photographs of (**a**) assembled hemispherical DRA with additional substrate (**b**) DRA in the anechoic chamber.

Figure 9a illustrates the utilized DRA prototype including the additional top substrates. With the aid of a 3D printer, the position of the DRA was outlined on the added top substrate in an attempt to avoid any misalignment. In addition, a small drop of glue was applied to bond the DRA to the top substrate. However, to minimize any potential gaps between the DRA and the Rogers substrate, the glue was applied to the outer DRA edge that was in contact with the substrate. Further, the top substrate was bonded to the ground plane using a thin, double-sided adhesive copper tape. The measurements were conducted using the E5071C mm-wave vector network analyzer, as well as the spherical near-field mm-wave measurement system (SNF-FIX-1.0) [30]. The reflection coefficient was measured using a 2.92 mm SMA.

**Figure 10:**
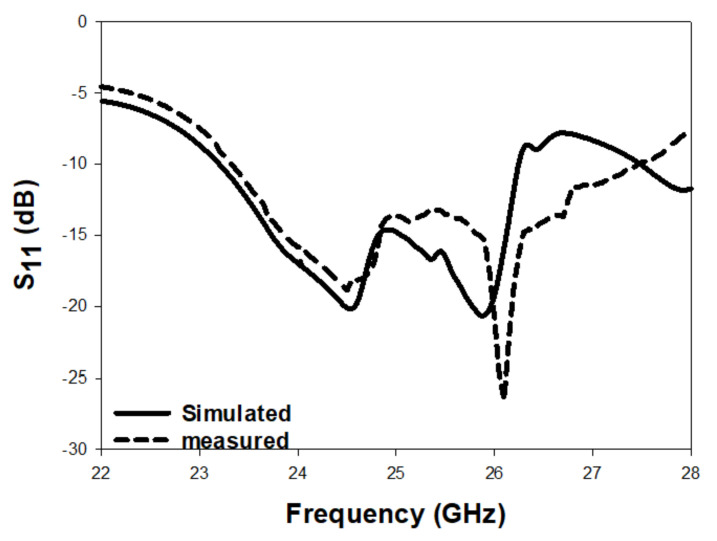
Reflection coefficient of a circularly polarised hemispherical DRA with added substrate between the DRA and feeding slot.

**Figure 11:**
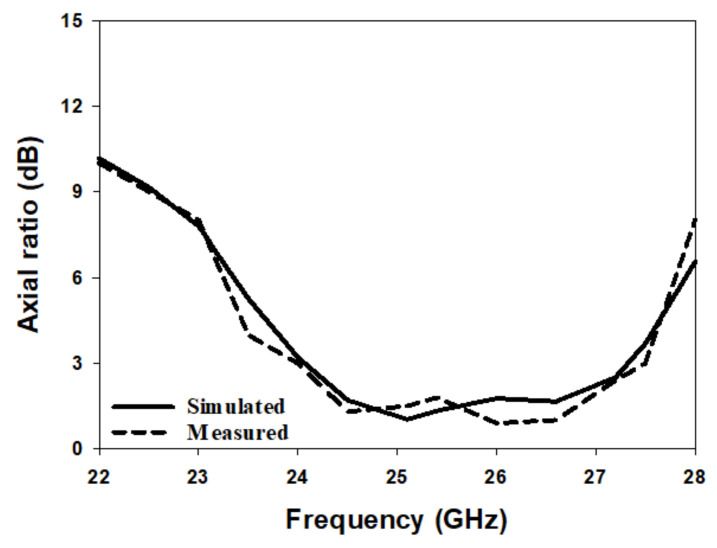
Simulated and measured axial ratio of a circularly polarised hemispherical DRA with added substrate between the DRA and feed.

**Figure 12:**
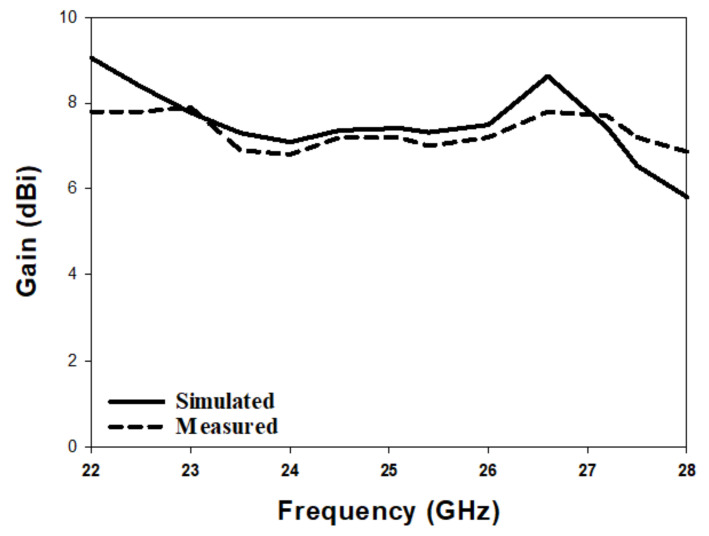
Simulated and measured gain of a circularly polarised hemispherical DRA with added substrate between the DRA and feed.

**Figure 13:**
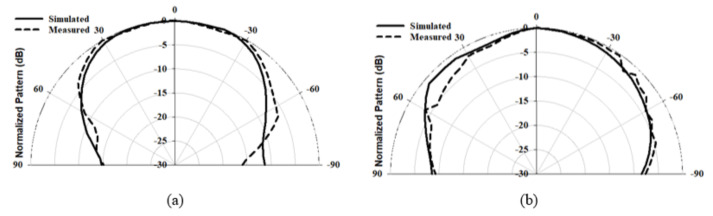
Radiation pattern of a hemispherical DRA with the added substrate at 24 GHz (**a**) ϕ = 0${}^{\circ }$ (**b**) ϕ = 90${}^{\circ }$.

**Figure 14:**
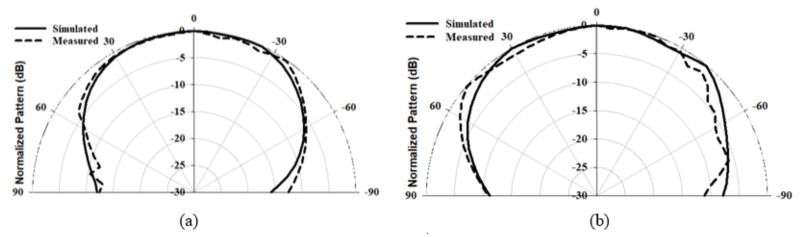
Radiation pattern of a hemispherical DRA with the added substrate at 26 GHz (**a**) ϕ = 0${}^{\circ }$ (**b**) ϕ = 90${}^{\circ }$.

Figure 10 illustrates the simulated and measured reflection coefficients, where a close agreement can be seen between the simulated and measured bandwidths of 13.2% and 14%, respectively. Once more, the impedance matching bandwidth was achieved by exciting the $TE_{311}$ and $TE_{112}$ resonance modes at 23 GHz and 26 GHz, respectively. In addition, the axial ratio is illustrated in Figure 11, where it can be observed that the achieved simulated and measured CP bandwidths were 11.8% and 12.1%, respectively. It should be noted that the impedance bandwidth extended from 21.2 GHz to 25.7 GHz, while the CP bandwidth extended from 23.8 GHz to 27.5 GHz. As a result, an overlap of ∼2 GHz was achieved between the two bandwidths, corresponding to ∼9%. Further, the achieved CP bandwidth was considerably wider than those reported in the literature for hemispherical DRAs. Figure 12 illustrates the simulated and measured broadside gain, with a close agreement in terms of the average of 7.5 dBi. Figure 13 and Figure 14 demonstrate a close agreement between the simulated and measured normalized radiation patterns of the excited resonance modes at 24 GHz and 26 GHz, respectively. The respective simulated and measured half-power beamwidths are 79° and 108° at 24 GHz, as well as 84° and 110° at 26 GHz, respectively. However, the marginal discrepancy between simulated and measured results is due to fabrication and experimental errors.

It should be noted that there are a couple of limitations that result from adding the top dielectric substrate. For instance, the DRA and substrate were assembled manually, which may be impractical at higher frequencies, e.g., 60 GHz and above. In addition, there is a limited choice of commercial substrates with the required combination of permittivity and thickness. Therefore, the next design proposes a technique where the DRA alignment and bonding are automated by integrating the DRA and the top substrate using 3D-printing technology.

## 4. Integrated Hemispherical DRA and Perforated Substrate

Since the optimum combination of substrate height and dielectric constant is commercially unavailable, a perforated top substrate is utilized where the effective dielectric constant can be adjusted by altering the diameter of the air-filled holes [31]. In addition, the design can be considerably simplified if an Alumina top substrate is utilized, since this facilitates the fabrication of an integrated DRA-substrate configuration without extra alignment and bonding measures. As reported in [32] for a linearly polarized DRA, such a configuration can be 3D-printed with air-filled holes in the Alumina substrate to ensure that an effective dielectric constant of $\varepsilon_{r2}$ = 3 is achieved using the predetermined optimum thickness of $h_{2}$ = 0.3 mm. The parameters of the DRA element and feed network are the same as those used in Section 2.

**Figure 15:**
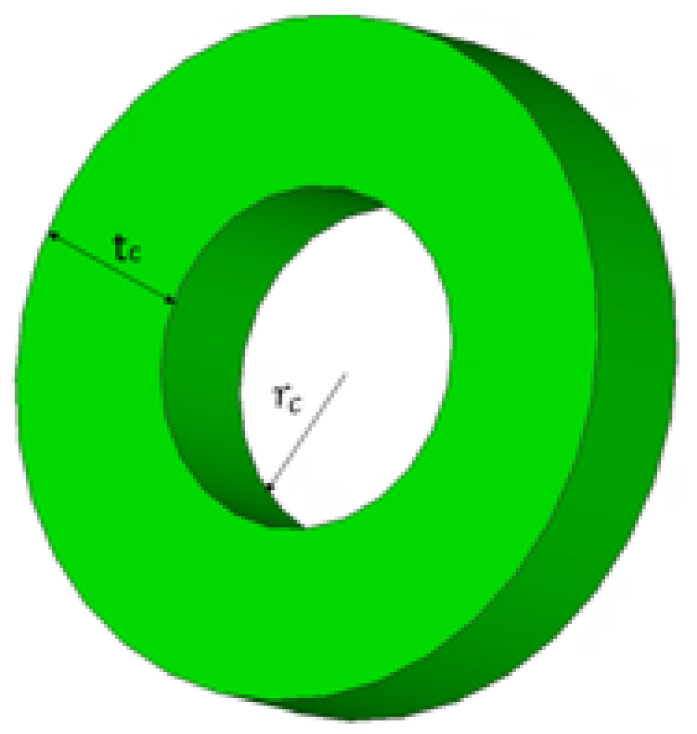
Unit cell of the perforated top substrate.

To obtain the optimal effective permittivity of the perforated substrate, a cylindrical unit cell was considered, with a radius of $r_{c}$, and a thickness of $t_{c}$ as illustrated in Figure 15 The height of the unit cell is the same as the thickness of the top substrate, i.e., 0.3 mm. In accordance with the theory of air-filled holes, tc determines the effective permittivity [33].

**Figure 16:**
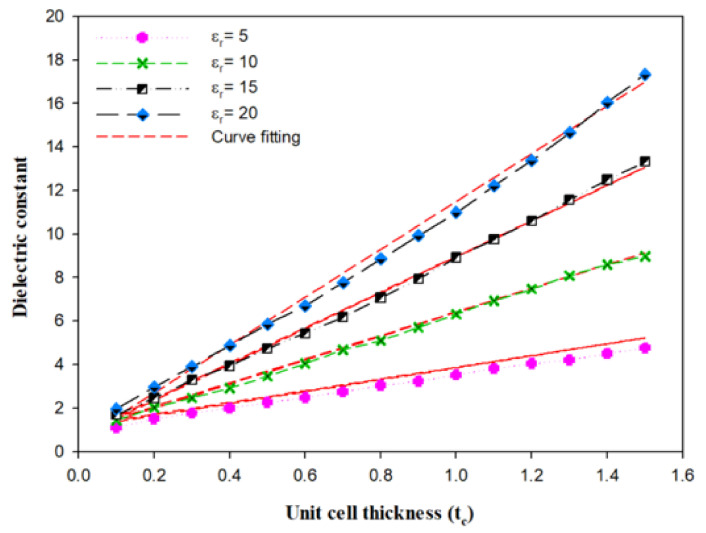
Effective relative permittivity of perforated hemispherical DRA [29].

**Figure 17:**
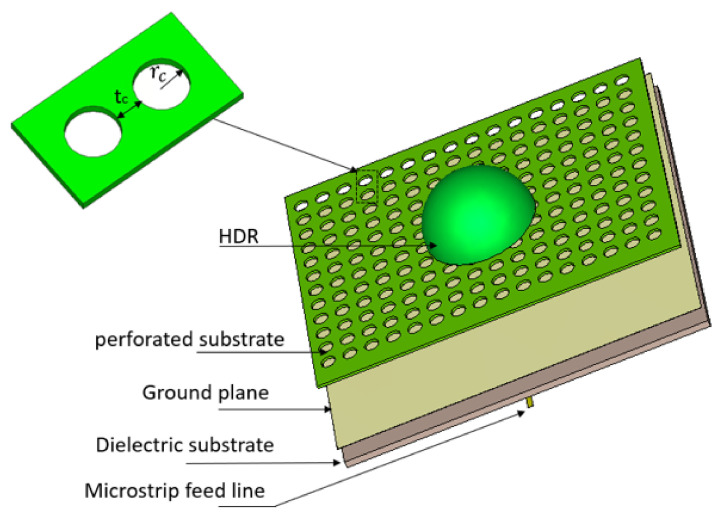
Geometry of the integrated hemispherical DRA and perforated top substrate.

The unit cell was designed using CST to determine the effective permittivity by placing it inside a waveguide. The perfect magnetic conducting (PMC) sides and perfect electric conducting (PEC) front and end surfaces were assumed. The effective permittivity of the unit cell can be extracted from the S-parameter by changing the material’s dielectric constant from 5 to 20, as demonstrated in Figure 16, where it can be observed that a linear relationship exists between the thickness of the unit cell and effective permittivity [34]. For example, when $\varepsilon_{r}$ = 5 or 20 and $t_{c}$ = 0.1 or 1.4 mm, the effective dielectric constant is $\varepsilon_{eff}$ = 1.3 and 4.7, respectively. For the sake of simplicity, to find the suitable radius and thickness of a unit cell, a curve-fitting formula was developed, as stated in the equation below:

(11) $$\varepsilon_{eff}=0.5t_{c}\varepsilon_{r}-0.1\varepsilon_{r}-0.15$$

from which it can be noted that the effective permittivity is determined by $t_{c}$. Figure 16 illustrates a close agreement between the original data extracted from the S-parameter and the curve-fitting results. The geometry of the integrate DRA and perforated substrate is demonstrated in Figure 17.

As mentioned in the previous section, when the dialectic constant of the top substrate is increased, the impedance bandwidth and resonance frequency are reduced; therefore, the perforated technique was introduced to overcome this challenge by reducing the effective permittivity of the integrated top Alumina substrate. The spacing ($t_{c}$) between the sides of adjacent air holes should be increased to reduce the effective permittivity, as shown in Figure 16. A perforated substrate with an effective dielectric constant of ∼3 was achieved by choosing the radius of the holes as $r_{c}$ = 0.5 mm, which corresponds to 0.08$\lambda_{0}$ and tc at 0.4 mm.

## 5. Experimental Setup and Results of Integrated DRA Configuration

**Figure 18:**
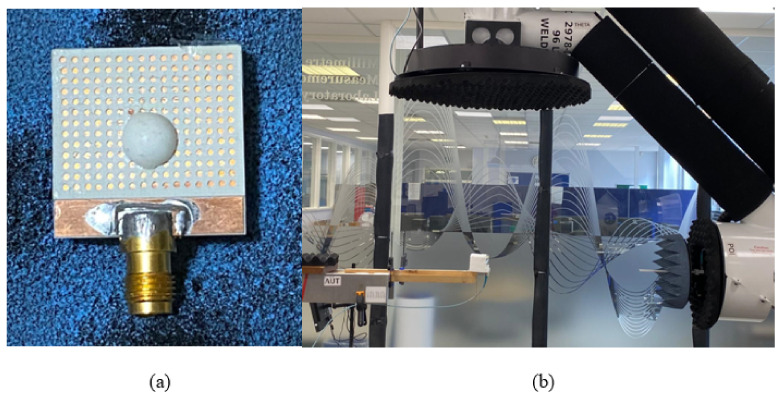
Integrated hemispherical DRA and a perforated substrate (**a**) Assembled (**b**) In the anechoic chamber.

**Figure 19:**
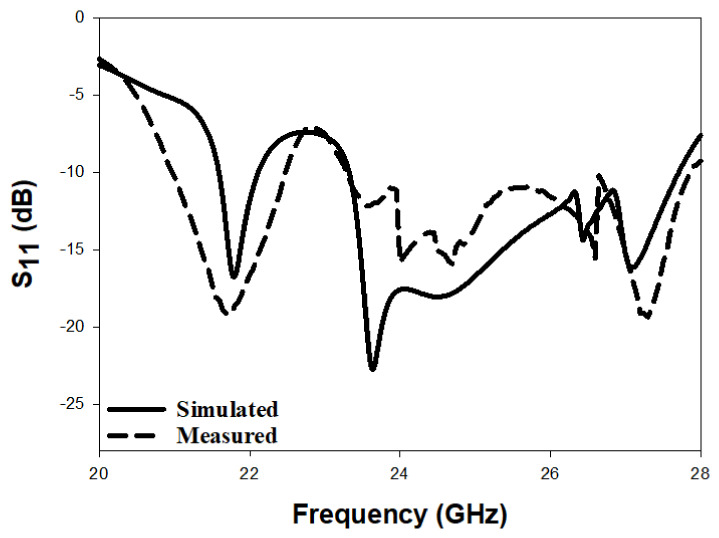
Reflection coefficient of a circularly polarised integrated hemispherical DRA and a perforated substrate.

Figure 18 illustrates the configuration of the integrated hemispherical DRA and perforated substrate. The DRA was fabricated using Alumina and integrated to the perforated Alumina substrate using 3D printing. The feeding structure was fabricated separately and was the same as that used in Section 3. Figure 19 presents the simulated and measured reflection coefficients that extend over the frequency ranges of 23.3–27.6 GHz and 23.2–27.8 GHz, respectively. These frequency ranges correspond to respective percentage bandwidths of 16.8% and 18% for the simulated and measured results. These results show that the simulated and measured reflection coefficient bandwidths are in good agreement.

**Figure 20:**
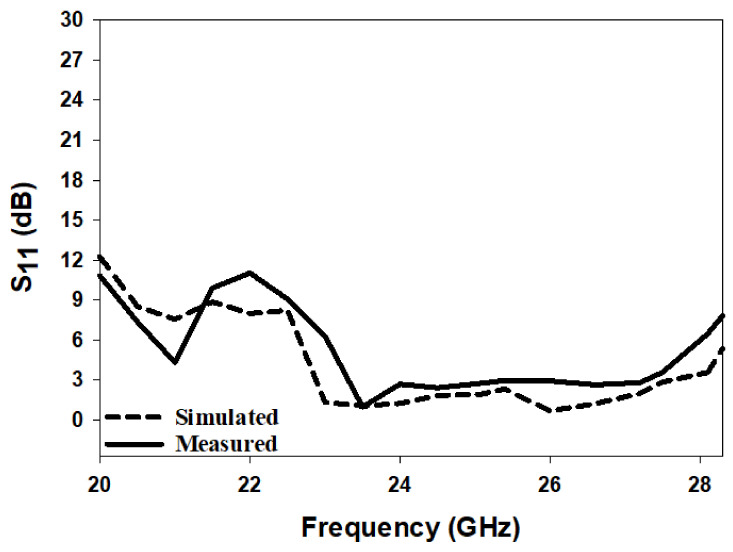
Axial ratio of a circularly polarised integrated hemispherical DRA and a perforated substrate.

**Figure 21:**
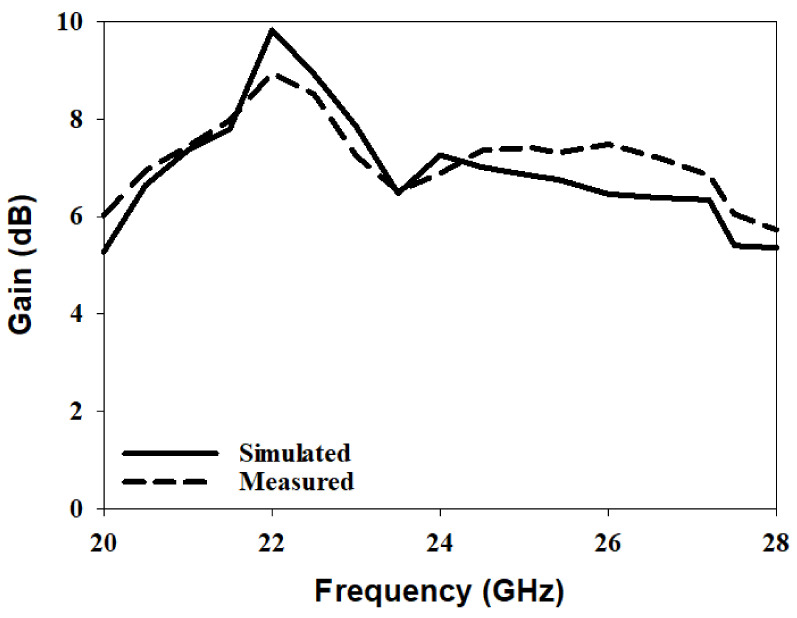
Broadside gain of a circularly polarised integrated hemispherical DRA and a perforated substrate.

**Figure 22:**
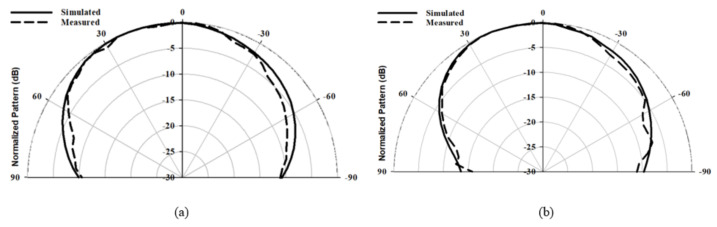
Radiation pattern of the integrated hemispherical DRA with perforated substrate excited in the $TE_{112}$ mode at 26 GHz (**a**) ϕ = 0${}^{\circ }$ (**b**) ϕ = 90${}^{\circ }$.

The radiation pattern, CP bandwidth, and gain were measured using the spherical near-field mm-wave measurement system (SNS-FIX-1.0). As demonstrated in Figure 20, the simulated and measured CP bandwidths were 16.2% and 18%, respectively, with an overlap between the CP and S11 bandwidths of 18%. Further, the achieved CP bandwidth was considerably wider than those reported in the literature for hemispherical DRAs. Figure 21 illustrates the simulated and measured broadside gain, with an average of 7.5 dBi and a close agreement between simulation and measurements. The simulated and measured broadside radiation patterns are presented in Figure 22 at the two principal plane cuts at 26 GHz, with close agreement between measurements and simulations. However, there is a marginal discrepancy between simulated and measured results owing to fabrication and experimental errors. In addition, any difference in the practical dielectric properties of the used materials also contributes to the difference between simulations and measurements.

The presented CP HDRA’s performance parameters, bandwidth, gain, efficiency, mode and AR, are listed in Table 1 and compared to those of different mmWave CP DRA configurations that have been reported in the literature. The proposed approach provides a wider AR bandwidth compared to those in the reported studies.

## 6. Conclusions

Three configurations of circularly polarized hemispherical DRA have been proposed, with a cross-slot feed utilized in each case. In the first design, the DRA was placed above the ground plane, and in the other two designs, the solid and perforated dielectric substrates were inserted between the DRA and the ground plane. In the absence of an additional substrate, respective impedance and AR bandwidths of 8.3% and 3.6% were achieved, with a gain of 6.5 dBi and an efficiency of 95%. Adding a Rogers substrate between the DRA and ground plane provided a significant increase in the impedance and CP bandwidths, to 14% and 12.1%, respectively, with an overlapping bandwidth of 8%, as well as reducing the impact of any DRA-feed misalignment. However, this configuration requires an adhesive material to firmly fix the DRA to the substrate. In addition, the permittivity and thickness of the substrates are determined by commercial availability. Therefore, a 3D-printed perforated Alumina substrate was utilized, where the air-filled holes were introduced and used to obtain the required effective dielectric constant. This resulted in an integrated DRA-perforated substrate configuration, which offers the flexibility to control the substrate parameters without being restricted to the commercially available substrates. In addition, the integrated DRA-perforated approach overcomes the limitations of using DRA assembly, alignment, and bonding. As a result, an optimum effective dielectric constant and height were achieved, which improved the CP bandwidth by increasing it from 12.1% to 18% when the solid top substrate was replaced by a perforated counterpart. The achieved bandwidths and gain were considerably higher than those reported in the literature for a hemispherical DRA.

## Figures and Tables

**Table 1:** Comparison between the performance of circularly polarized DRAs.

Ref.	DRA Shape	Frequency (GHz)	Bandwidth (%)	Gain (dB)	Efficiency (%)	Mode	Axial Ratio (%)
[17]	Rectangular	20/30	6.4/12.8	6.6/8.2	NM	$TE_{111}$	5.2/4.1
[18]	Rectangular	26	36.5	12.5	90	$TE_{119}$	13.75
[19]	Flower	30	33.8	9.5	NM	NM	5
[21]	Cylindrical	60	24.2	5.5	92	$HEM_{11\delta }$	4
[22]	Cylindrical	60	11.8	11.43	84	$HEM_{11\delta }$	15.9
[35]	Rectangular	30	16.48	12.7	NM	$TE_{11\delta }$	1.1
[36]	Rectangular	24	15.06	7.9	NM	$TE_{117}$	5.8
[37]	Cylindrical	26	26	6.6	NM	$TE_{412}$	1.35
This work	Hemispherical	26	18	7.5	95	$TE_{112}$	18

## Data Availability

Not applicable.

## References

[1] Buerkle A., Sarabandi K., Mosallaei H. (2005). Compact slot and dielectric resonator antenna with dual-resonance, broadband characteristics. IEEE Trans. Antennas Propag..

[2] Junker G.P., Kishk A.A., Glisson A.W. (1996). Input impedance of aperture-coupled dielectric resonator antennas. IEEE Trans. Antennas Propag..

[3] Loconsole A.M., Francione V.V., Portosi V., Losito O., Catalano M., Di Nisio A., Attivissimo F., Prudenzano F. (2021). Substrate-Integrated Waveguide Microwave Sensor for Water-in-Diesel Fuel Applications. Appl. Sci..

[4] Ren Y.-J., Chang K. (2006). New 5.8-GHz circularly polarized retrodirective rectenna arrays for wireless power transmission. IEEE Trans. Microw. Theory Tech..

[5] Kumar R., Chaudhary R.K. (2018). Investigation of higher order modes excitation through F-shaped slot in rectangular dielectric resonator antenna for wideband circular polarization with broadside radiation characteristics. Int. J. Microw. Comput.-Aided Eng..

[6] Wang K.X., Wong H. (2015). A Circularly Polarized Antenna by Using Rotated-Stair Dielectric Resonator. IEEE Antennas Wirel. Propag. Lett..

[7] Yang M., Pan Y., Yang W. (2018). A Singly Fed Wideband Circularly Polarized Dielectric Resonator Antenna. IEEE Antennas Wirel. Propag. Lett..

[8] Lin C.C., Sun J.S. (2015). Circularly Polarized Dielectric Resonator Antenna Fed by Off-Centered Microstrip Line for 2.4-GHz ISM Band Applications. IEEE Antennas Wirel. Propag. Lett..

[9] Gupta S., Sharma A., Das G., Gangwar R.K., Khalily M. (2019). Wideband Circularly Polarized Dielectric Resonator Antenna Array with Polarization Diversity. IEEE Access.

[10] Kumar R., Chaudhary R.K. (2016). A Wideband Circularly Polarized Cubic Dielectric Resonator Antenna Excited with Modified Microstrip Feed. IEEE Antennas Wirel. Propag. Lett..

[11] Khalily M., Rahim M.K.A., Kishk A.A. (2012). Planar Wideband Circularly Polarized Antenna Design with Rectangular Ring Dielectric Resonator and Parasitic Printed Loops. IEEE Antennas Wirel. Propag. Lett..

[12] Ren X., Liao S., Xue Q. (2019). A Circularly Polarized Spaceborne Antenna with Shaped Beam for Earth Coverage Applications. IEEE Trans. Antennas Propag..

[13] Pan Y., Leung K.W. (2010). Wideband circularly polarized trapezoidal dielectric resonator antenna. IEEE Antennas Wirel. Propag. Lett..

[14] Chaudhary P., Ghodgaonkar D.K., Gupta S., Jyoti R., Mahajan M.B. (2020). Compact Circularly Polarized Triband Staired Rectangular Dielectric Resonator Antenna Using Single and Dual Sections WPD with Phase Shifter For Navigation Satellite Applications. Microw. Opt. Technol. Lett..

[15] Fakhte S., Oraizi H., Karimian R. (2014). A Novel Low-Cost Circularly Polarized Rotated Stacked Dielectric Resonator Antenna. IEEE Antennas Wirel. Propag. Lett..

[16] Ittipiboon A., Roscoe D., Mongia R., Cuhaci M. A Circularly Polarized Dielectric Guide Antenna with a Single Slot Feed. Proceedings of the IEEE Symposium on Antenna Technology and Applied Electromagnetics.

[17] Xu H., Chen Z., Liu H., Chang L., Huang T., Ye S., Zhang L., Du C. (2022). Single-Fed Dual-Circularly polarized Stacked Dielectric Resonator Antenna for K/Ka-Band UAV Satellite Communications. IEEE Trans. Veh. Technol..

[18] Abdulmajid A.A., Khamas S., Zhang S. (2020). Wide bandwidth high gain circularly polarized millimetre-wave rectangular dielectric resonator antenna. Prog. Electromagn. Res..

[19] Kesavan A., Al-Hassan M., Mabrouk I.B., Denidni T.A. (2021). Wideband circular polarized dielectric resonator antenna array for millimeter-wave applications. Sensors.

[20] Akbari M., Gupta S., Farahani M., Sebak A.R., Denidni T.A. (2016). Gain enhancement of circularly polarized dielectric resonator antenna based on FSS superstrate for MMW applications. IEEE Trans. Antennas Propag..

[21] Lai Q., Fumeaux C., Hong W., Vahldieck R. (2010). 60 GHz aperture-coupled dielectric resonator antennas fed by a half-mode substrate integrated waveguide. IEEE Trans. Antennas Propag..

[22] Sun Y.-X., Leung K.W. (2018). Circularly polarized substrate-integrated cylindrical dielectric resonator antenna array for 60 GHz applications. IEEE Antennas Wirel. Propag. Lett..

[23] Wa L.K., Kuen N.H. (2005). The slot-coupled hemispherical dielectric resonator antenna with a parasitic patch: Applications to the circularly polarized antenna and wide-band antenna. IEEE Trans. Antennas Propag..

[24] Leung K.W., Ng H.K. (2003). Theory and experiment of circularly polarized dielectric resonator antenna with a parasitic patch. IEEE Trans. Antennas Propag..

[25] Qian Z., Leung K., Chen R.-S. (2004). Analysis of circularly polarized dielectric resonator antenna excited by a spiral slot. Prog. Electromagn. Res..

[26] Lam H., Leung K. (2006). Analysis of U-slot-excited dielectric resonator antennas with a backing cavity. IEE Proc.-Microwaves Antennas Propag..

[27] Leung K. (2002). AEfficient computation for the general solution of a slot loaded by a hemispherical dielectric and/or backing cavity. IEEE Trans. Antennas Propag..

[28] Chaudhary R.K., Kumar R., Chowdhury R. (2021). Circularly Polarized Dielectric Resonator Antennas.

[29] Alanazi M.D., Khamas S.K. (2022). Wideband mm-Wave Hemispherical Dielectric Resonator Antenna with Simple Alignment and Assembly Procedures. Electronics.

[30] UKRI National Millimetre Wave Facility. https://www.sheffield.ac.uk/mm-wave/.

[31] Liang M., Ng W.R., Chang K., Gbele K., Gehm M.E., Xin H. (2014). A 3-D Luneburg lens antenna fabricated by polymer jetting rapid prototyping. IEEE Trans. Antennas Propag..

[32] Xia Z.X., Leung K.W., Lu K. (2019). 3-D-printed wideband multi-ring dielectric resonator antenna. IEEE Antennas Wirel. Propag. Lett..

[33] Smith D.R., Vier D.C., Koschny T., Soukoulis C.M. (2005). Electromagnetic parameter retrieval from inhomogeneous metamaterials. Phys. Rev. E.

[34] Chen X., Grzegorczyk T.M., Wu B.-I., Pacheco J., Kong J.A. (2004). Robust method to retrieve the constitutive effective parameters of metamaterials. IEEE Antennas Wirel. Propag. Lett..

[35] Chu H., Guo Y.-X. (2016). A novel approach for millimeter-wave dielectric resonator antenna array designs by using the substrate integrated technology. IEEE Trans. Antennas Propag..

[36] Bansal A., Vaish A. (2016). Deminiaturized mode control rectangular dielectric resonator antenna. Prog. Electromagn. Res..

[37] Zhao G., Zhou Y., Wang J.R., Tong M.S. (2022). A circularly polarized dielectric resonator antenna based on quasi-self-complementary metasurface. IEEE Trans. Antennas Propagationl.

